# The C3HeB/FeJ mouse model recapitulates the hallmark of bovine tuberculosis lung lesions following *Mycobacterium bovis* aerogenous infection

**DOI:** 10.1186/s13567-017-0477-7

**Published:** 2017-11-07

**Authors:** Mélodie Bouté, Florence Carreras, Christelle Rossignol, Emilie Doz, Nathalie Winter, Mathieu Epardaud

**Affiliations:** 1 INRA, Université de Tours, UMR 1282, Infectiologie et Santé Publique, Nouzilly, France; 2 Present Address: U1019, UMR8204, Université Lille, CNRS, INSERM, CHU Lille, Institut Pasteur de Lille, Lille, France

## Abstract

Achieving the control of bovine tuberculosis (bTB) would require the discovery of an efficient combined immunodiagnostic and vaccine strategy. Since in vivo experiments on cattle are not ethically and economically acceptable there is a need for a cost-effective animal model capable of reproducing, as closely as possible, the physiopathology of bTB to (i) better characterize the cellular and molecular features of bTB immunopathogenesis and (ii) screen preclinical vaccine candidates. To develop such a model, we focused on the C3HeB/FeJ Kramnik’s mouse forming hypoxic, encapsulated granulomas with a caseous necrotic center following *Mycobacterium tuberculosis* infection. Our work represents the first investigation on C3HeB/FeJ interaction with *M. bovis*, the main agent of bTB. Detailed histopathological analysis of C3HeB/FeJ lung lesions development following aerogenous *M. bovis* infection unraveled a bimodal evolution of the pathology. The C3HeB/FeJ recapitulated all the hallmarks of classical bovine lung granulomas but also developed, to some extend, lethal necrotic large lesions characterized by high mycobacterial and neutrophil load, and an inefficient collagen-driven lesion encapsulation. Interestingly these rapidly invasive pneumonia lesions, occurring in a constant percentage of the mice, shared all features with some exacerbated lung lesions that we and others have observed in lungs of cattle naturally or experimentally infected with *M. bovis*. Together, our findings demonstrate the relevance of the C3HeB/FeJ mouse as a comprehensive model to study bTB immunopathology that could be used for further vaccine therapies in the future.

**Electronic supplementary material:**

The online version of this article (10.1186/s13567-017-0477-7) contains supplementary material, which is available to authorized users.

## Introduction

Bovine tuberculosis (bTB), a chronic disease of cattle caused by *Mycobacterium bovis*, is a zoonosis that represents a significant economic animal health problem with a worldwide estimated prevalence of infection of 9% and an annual cost of 3 billions $ [1–3].

French national control programs rely on fast and cost-effective diagnostic methods and mandatory slaughter of positive herds to control and eradicate the disease (WHO report, 2011) [4]. Thanks to such costly programs—highest budget line of the Department of Agriculture estimated around 30 millions €/year [5, 6]—maintaining a national prevalence below 0.1% is now achieved which identifies France as an Officially bTB Free country.

The eradication of the disease is complicated by the fact that *M. bovis* can infect a wide range of mammals and thus can establish reservoirs in wildlife, including badger in the UK, wild boar and deer in France and Spain and White-tailed deer in the US. All these species are involved in the spreading of infection to cattle [7]. Furthermore, recent results suggest that *M. bovis* is also able to persist within environmental samples in areas where infected cattle or wildlife were detected and eliminated [8]. These bTB features render eradication solely based on diagnosis and slaughter highly challenging.

Taken together these data point out the necessity to develop an effective vaccine with vaccine-compatible diagnosis tests to distinguish vaccinated from infected animals (DIVA) [9].

To fulfill this objective there is a need, first, to better characterize the cellular and molecular processes involved in bTB immune-pathogenesis and thus to focus on its hallmark: the granuloma. This organized and dynamic structure is known to be a key element in the transmission process as well as the host resistance to the disease [10, 11].

Granulomas are shaped by the structured aggregation of immune cells in cascade and are classically composed, at their mature stage, by macrophages, foamy and epithelioid, scattered multinucleated Langhans giant cells (MGCs) and neutrophils surrounded by a ring of lymphocytes coated by a collagen dense fibrotic capsule [10]. These structures have been largely documented in human TB (TB) and more scarcely in naturally or experimentally infected cattle and calves [12, 13]. The presence in bTB infected cattle granulomas of specific cell populations such as foamy macrophages, epithelioid cells, MGCs and neutrophils suggest that, similarly to the situation in humans, these cells drive the outcome of cattle immune response to *M. bovis* infection.

One additional critical step to consider in TB resistance relies on the granuloma encapsulation process. This mechanism involves the production of fibrotic, collagen rich, connective tissues around the granuloma, essential for controlling growth and spreading of mycobacteria.

Consequently, careful study of the composition and dynamics of the lesions could highlight fundamental host defenses mechanisms on which to base an efficient vaccine strategy. However conducting such analysis directly in cattle, the target species, would be ethically and economically unacceptable. By contrast, the use of small animal models represent a cost-effective means to evaluate the immunopathology process to further design vaccination strategies. To reach this goal, the C3HeB/FeJ mouse (Ipr1 deficient, also known as the Kramnik’s mouse, [14, 15]) represents an attractive model because it recapitulates the essential hallmark of TB histopathology. The C3HeB/FeJ mice share a common origin with the C3H/HeJ mice, commonly called C3H, but don’t carry their spontaneous TLR4 mutation (TLR4^lps−d^) [16]. Following infection with *M. tuberculosis*, C3HeB/FeJ develop typical granuloma i.e.: hypoxic, collagen encapsulated, caseous necrotic pulmonary lesions loaded with intracellular bacilli in the periphery and extracellular bacilli in the central necrotic core [17, 18]. On the contrary, classical TB resistant mouse models such as BALB/c or C57Bl/6 show limited features of TB physiopathology [19, 20]. The high susceptibility of C3HeB/FeJ mice to TB infection was associated with the identification of the “super-susceptibility to tuberculosis-1” (sst1) locus on chromosome 1. The sst1 allele was linked to the weak capacity to control TB multiplication and the occurrence of caseous necrotic lesions in the lung (reviewed in [21]). Further genetics investigations allowed the identification of the “intracellular pathogen resistance 1” (Ipr1), an isoform of the “interferon-inducible-75” gene (Ifi75), responsible for most of these features [21, 22].

To our knowledge, the outcome of *M. bovis* infection in the C3HeB/FeJ mouse strain has not been tested. Based on the description of its sensitivity to human TB, caused by *M. tuberculosis* strains, we made the hypothesis that the C3HeB/FeJ mouse strain could represent a relevant model for further in vivo studies of the bTB disease in cattle. However, this first required that we established that these mice could reproduce the main histopathological features of bTB development in cattle.

In the present study we showed that, following *M. bovis* intranasal infection, the C3HeB/FeJ mouse model recapitulated the classical granuloma found in the lung of cattle following aerogenous, natural or experimental, infection with *M. bovis*. Furthermore, we pointed out its capacity to alternatively develop early extended granulomatous lesions, with multicentric caseous necrotic core rich in neutrophils highly loaded with bacilli but inefficiently encapsulated, akin to the invasive necrotizing granuloma lesions described in the lung of cattle naturally and experimentally infected [23, 24]. Furthermore, using histopathology, these lesions could also be compared to some exacerbated lung lesions, recently described in naturally infected cattle [25]. Thus, the presence of these different types of lesions in the C3HeB/FeJ mouse model, following aerogenous infection with *M. bovis*, was clearly relevant to bovine TB physiopathology attesting the significance of this model.

## Materials and methods

### Bovine lung histology

Slides from PFA fixed and paraffin-embedded lung nodules from cattle naturally infected (Charolais breed, Côte d’Or, France) with *Mycobacterium bovis* were provided by Dorothée Virieux-Watrelot (VetAgroSup, Lyon, France) and subsequently processed in our laboratory for haematoxylin and eosin (H&E) staining or Masson-Goldner’s Trichrome (Goldner’s Trichrome) staining following the same protocols as used for C3HeB/FeJ mice tissues (see below).

### Ethics statement and mouse treatments

Experimental protocols complied with French law and EEC regulations for the care and use of laboratory animals and were carried out under Authorization for Experimentation on Laboratory Animals Number B-37-201. Our animal protocol (#2012-06-14) was approved by the “Val de Loire” Ethics Committee for Animal Experimentation (CEEA VdL) and was registered with the French National Committee for Animal Experimentation. C3HeB/FeJ mice, originally purchased from Jackson Laboratories (Bar Harbor, ME, USA), and C57BL/6 mice, originally purchased from Janvier Laboratories (Le Genest-Saint-Isle, France), were raised in our animal facility. Male and Female mice aged 6–8 weeks were housed in a bio-safety level III animal facility (PlateForme d’Infectiologie Experimentale: PFIE UE-1277 INRA Centre Val de Loire Nouzilly, France) and maintained with sterile bedding, water and mouse chow. Specific pathogen-free status was verified by testing sentinel mice housed within the colony.

Terminal end-point was determined by a weight loss of 15% or more of their pre-experimental body weight detected over a 2 day period and accompanied by other clinical signs comprising isolation, inactivity, hunched posture, very greasy and ruffled fur.

### Bacteria

*Mycobacterium bovis* AF2122/97, a fully virulent strain isolated from lung caseous lesions of a tuberculin test reactor cow in Great Britain in 1997, has been entirely sequenced and its genome annotation recently updated and is the reference model for *M. bovis* for bovine TB investigation [26, 27]. Thus, the benchmark AF2122/97 strain was used for our intranasal infections of mice as previously described [28]. The bacteria were originally grown in 7H9 medium supplemented with 4.16 g/L pyruvic acid (Sigma), 10% v/v ADC (Becton–Dickinson) and 0.05% v/v Tween 80 (Sigma). Bacteria were harvested at mid-exponential growth phase and frozen at −80 °C until use. CFU were counted after plating dilutions on Middlebrook 7H11 agar (Becton–Dickinson) supplemented with 4.16 g/L pyruvic acid (Sigma) and with OADC (ADC supplemented with 0.05% oleic acid, Becton–Dickinson).

### Intranasal infection

C3HeB/FeJ mice were infected with *M. bovis* strain AF2122/97 following a protocol already settled on Balb/C mice by Logan et al. [28] and routinely used with *M. tuberculosis* in our laboratory. Mice were anesthetized by intraperitoneal (i.p.) injection of ketamine/xylasine cocktail and received 200 CFU of *M. bovis* under 20 μL in each nostril. Five mice sacrificed the following day allow the evaluation of the number of CFU implanted in the lungs with an average of 134 bacteria on day one post-inoculation.

### Enumeration of bacterial load of lungs and other tissues

At the time of sacrifice, whole lung, mediastinal lymph node, spleen and liver were aseptically removed and used for bacterial enumeration. The number of viable organisms was determined by plating serial dilutions of whole tissues disrupted with ceramic beads (Lysing Matrix D, MP Biomedicals Europe, Illkirch, France) in phosphate buffered saline (PBS), in a Ribolyser apparatus (Fastprep-24, MP Biomedicals), in accordance with the manufacturer’s instructions. Appropriate dilutions were plated on 7H11 plates supplemented with 4.16 g/L pyruvic acid (Sigma) and with OADC (Becton–Dickinson). Colonies were counted after at least 21 days of incubation at 37 °C.

### Histopathology

At necropsy, whole lungs and mediastinal lymph node were transferred for fixation in 4% paraformaldehyde (PFA) in PBS for 48 h then dehydrated and kept in 70% ethanol before embedment in paraffin wax until being processed for histopathological assessment. Whole tissue representative series of 5 μm-tick tissue sections were mounted on glass slides, deparaffinized and stained either with: haematoxylin and eosin, Ziehl-Neelsen (ZN) for the detection of acid-fast bacilli (AFB) or Masson-Goldner’s Trichrome for the detection of collagen.

Sections stained with H&E, ZN or Masson-Goldner’s Trichrome were visualized using a Nikon Eclipse 80i microscope with DS-Ri2 camera controlled by the NIS-Elements D software package (Nikon, Instruments Inc., Tokyo, Japan).

### Masson-Goldner’s trichrome staining

Collagen was stained in mouse lung tissue sections using a Goldner’s Trichrome with light green Kit (DiaPath, MM France, France) following the manufacturer’s recommendations. Briefly, tissue sections were dewaxed histosol and rehydrated through a graded alcohol series. Sections are briefly rinsed in water and slides are stained with Weigert’s iron hematoxylin mix and washed in tap water and incubated with alcoholic picric acid. After washing in distilled water, sections are stained with Ponceau acid fuchsin and without washing the sections were covered by a phosphomolybdic acid solution and without washing the slides are drained and light green is added. Finally without washing the sections a quick dehydration in ethanol and clearing in histosol (thermo electron, USA), the slides are mounted in Eukitt quick mounting medium (ORSATEC GNBH, Germany).

### Immunohistochemical staining

Tissue sections were processed for immunohistochemical staining of neutrophils. Ag retrieval step was performed by autoclaving tissue sections in 10 mM citrate buffer pH 6.1 for 20 min at 121 °C. Endogenous peroxidase was inhibited using a 1:100 dilution of 30% hydrogen peroxide (Sigma) in methyl alcohol for 30 min at room temperature. Washing steps were performed with tap water. Nonspecific binding sites were blocked with a 20-min incubation in 20% normal goat serum and 30% FCS (GIBCO). Immunohistochemical (IHC) detection of neutrophils was performed using a purified rat anti-mouse Ly-6G Ab (Clone 1A8, IgG2a, #551459; BD Biosciences) at a final concentration of 1.25 μg/mL. Immunolabeling the sections by incubation with this primary Ab for 60 min at room temperature was followed by a 30-min incubation with the universal immunoperoxidase polymer, anti-mouse N-Histofine (N-Histophine Simple Stain MAX PO (M), Nichirei Biosciences, Microm Microtech France). Enzymatic activity was revealed using diaminobenzidine (LabVision). Each step was followed by washes with 1% BSA and 0.05% Tween 20 in PBS. Sections were counterstained with a Harris hematoxylin solution. Control staining was performed with the use of purified rat IgG2a isotype control Ab (#553927, BD Biosciences) in place of the primary Ab.

To visualize hypoxic regions in lung tissues, the pimonidazole immunohistochemical staining technique was used. Mice were injected i.p. with 60 mg/kg pimonidazole hydroxychloride 1.5 h before sacrifice, and lungs and kidney were harvested, fixed, and stained with the Hypoxyprobe staining kit (Chemicon, Burlington, MA, USA) according to the manufacturer’s instructions. C3HeB/FeJ uninfected lung served as negative control while kidney sections containing hypoxic renal tubular cells served as positive control.

### Histological lesion-type determination, morphometric analysis of the lesions and quantification of the Ziehl^+^ areas

For each individual animal (*n* = 5 to 8 per time point), a total of 8 H&E-stained slides representative of the whole lung were scanned on a 3DHISTECH scanner (Sysmex). Representative slides for each mice were examined blindly by trained pathologists to determinate the type of lesions presents within the tissues.

H&E-stained slides (8 per animal) of all five lung lobes from individual animals were further quantified with Fiji software using an image J Macro that we developed [29]. The data were expressed as a ratio of lesion area to the total lung area (mean ± s.e.m.).

Ziehl-stained slides representatives of the total lung of each individual were quantified with Fiji software using an Image J Macro that we developed based on the high contrast of the mycobacteria staining. The data were expressed as a ratio of Ziehl^+^ area to the total lesion area (mean ± s.e.m.).

### Statistical analysis

Statistical analyses were carried out using GraphPad Prism 5 (GraphPad Software Inc., San Diego, CA, USA).

Correlation between bacterial load and percentage of total body weight loss was assessed by non-parametrical analysis applying the Spearman rank correlation (Spearman’s correlation indexes: r and *p* value are provided).

Comparison analysis of the percentage of total body weight loss or percentage of total Ziehl^+^ area for groups of mice carrying different lesion-types were performed using the Mann–Whitney test.

## Results

### Histopathology of tuberculous lesions from bovine naturally infected with *Mycobacterium bovis*

In several reports of natural or experimental aerogenous *M. bovis* infection in cattle, histopathological analysis of lung lesions was carefully recorded. We decided to compile all these data in Table 1 (adapted from [23, 24]) to reveal the presence, in the target species, of different lesions types with heterogeneous characteristics.

**Table 1:** Histopathology of cattle lung lesions following aerogenous infection with *M. bovis*. Adapted from Rhyan and Saari [32]; Cassidy et al. [13, 14]; Menin et al. [33]

Histopathology of cattle lung lesions following aerogenous infection with *M. bovis*	Classical granuloma	Invasive necrotizing granuloma	Chronic lymphocytic « cuffing » pneumonia
Core			
Central caseous necrosis of neutrophil origin	+	++ *Multifocal/invasive*	Inflammatory cells infiltrate perivascular or close to the bronchioles
Extra-cellular bacilli within necrotic core	++	+++	
Mineralization	±	±	
Mantle			
Intact and degenerated neutrophils	++	+++	**−**
Macrophages (activated & foamy)	++	++	+
Epitheliod macrophages	++	++	+
Multinucleated giant cells (MGC)	++	+	−
Lymphocytes	+	+	+++
Cellular bacilli within macrophages and neutrophils	++	+++	ND
Periphery			
Fibrous encapsulation (Collagenous connective tissue)	+++	±	−
Time of first appearance (post-infection day) [13]	*28* (Circumscribing fibrosis)	*14* (Large, multifocal, numerous bacilli)	*7*
Bacterial load (CFU)	+	+++	±
Satellite aggregations: neutrophils intact or degenerated	+	++	−

**Figure 1:**
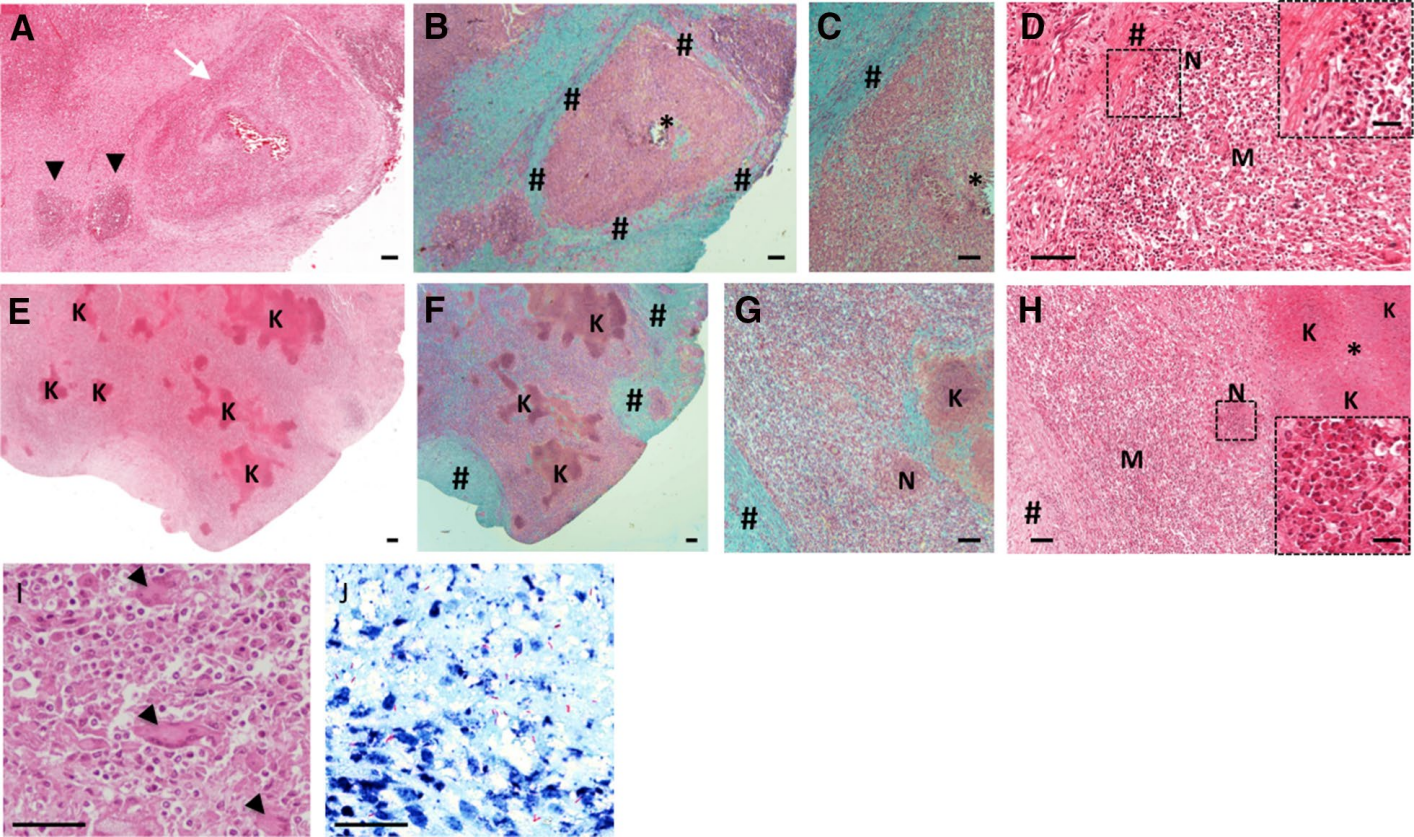
**Lung lesion from cattle naturally infected with** ***M. bovis.*** (**A**–**B**) H&E (**A**) and Masson-Goldner’s Trichrome (**B**) staining of a typical tuberculous classical granuloma lesion (white arrow) with central mineralization and peripheral fibrous capsule surrounded by satellites smaller lesions (black arrows). *: caseous necrosis, #: fibrous capsule. **C**, **D** Higher magnification of **A** and **B** showing an intact neutrophil area (“N” & Zoom) below the fibrous capsule (#) interspersed by foamy and epitheliod macrophages (“M”). **E**,** F** H&E (**E**) and Masson-Goldner’s Trichrome (**F**) staining of extended lesion with abundant caseous necrosis and karyorrhectic debris areas (“K”) but low discontinuous fibrous capsule (#), representative of an invasive necrotizing granuloma. **G**,** H** Higher magnification of E&F showing an example of a satellite aggregate of necrotic neutrophil (“N”, zoomed in (**H**)) next to the karyorrhectic debris (“K”) and caseous necrotic rich area (*). **I** High magnification of a foamy macrophages and epitheliod cell dense area containing multinucleated Langhans giant cells (black arrows). **J** Ziehl staining, high magnification of a central necrotic area showing extracellular bacilli within cellular debris. Scale bars: 25 μm (D-Zoom & H-Zoom); 50 μm (**I** and **J**); 100 μm (**C**, **G**, **D**, **H**); 200 μm (**A**, **B**, **E**).

In order to reinforce this bibliographic analysis, we also conducted an histopathological analysis in lungs sections from naturally infected cattle collected from *M. bovis* positive biopsies (Charolais breed, Côte d’Or, France) by H&E and Ziehl-staining (see representative sections of granuloma in Figure 1). Histopathological examination of the lung microscopic lesions revealed typical caseo-necrotic granulomas with central mineralization and peripheral fibrosis with nearby satellite smaller lesions (Figures 1A and B). This typical lesion represented a fully encapsulated tuberculous granuloma, as previously described (Table 1), characterized by a central necrotic core, caseous and mineralized (Figures 1A and B) surrounded by epithelioid and foamy macrophages and neutrophil clustering nearby the peripheral fibrotic capsule (Figures 1C and D).

Histological observations also revealed the presence of large and irregular lesions with multiple centers of caseous necrosis and sometimes mineralization (Figure 1E). These lesions displayed numerous islands of karyorrhectic debris with caseous necrosis and minimal mineralization surrounded by epithelioid macrophages and some multinucleated giant cells (Figures 1E, H and I). Moreover, these lesions presented a limited degree of circumscribing fibrosis and only few scattered lymphocytes (Figures 1F and G), On the other hand they contained many neutrophils intact (scattered) or degenerated (small aggregates) (Figure 1H) and Ziehl staining of the karyorrhectic areas showed the numerous extracellular bacilli within the necrotic debris (Figure 1J). Moreover, careful examination of the necrotic areas indicated the neutrophil origin of the karyorrhectic debris. Thus these lesions displayed many similarities with those observed in the lung of *M. bovis* infected cattle after natural infections as reported by Menin et al. or experimental exposure as reported by Cassidy et al. [23, 25]. They were also similar to invasive necrotizing granulomas described by Rhyan and Saari [30] (Table 1). In agreement with these previous reports, we also propose that during bTB development in cattle, large multifocal caseous lesions are formed by aggregation of neutrophils around mycobacteria and their subsequent degeneration into necrotic cores, and that they are poorly encapsulated (Figures 1E–J).

### Bacterial replication and associated morbidity in C3HeB/FeJ mice following intranasal infection with *M bovis*

**Figure 2:**
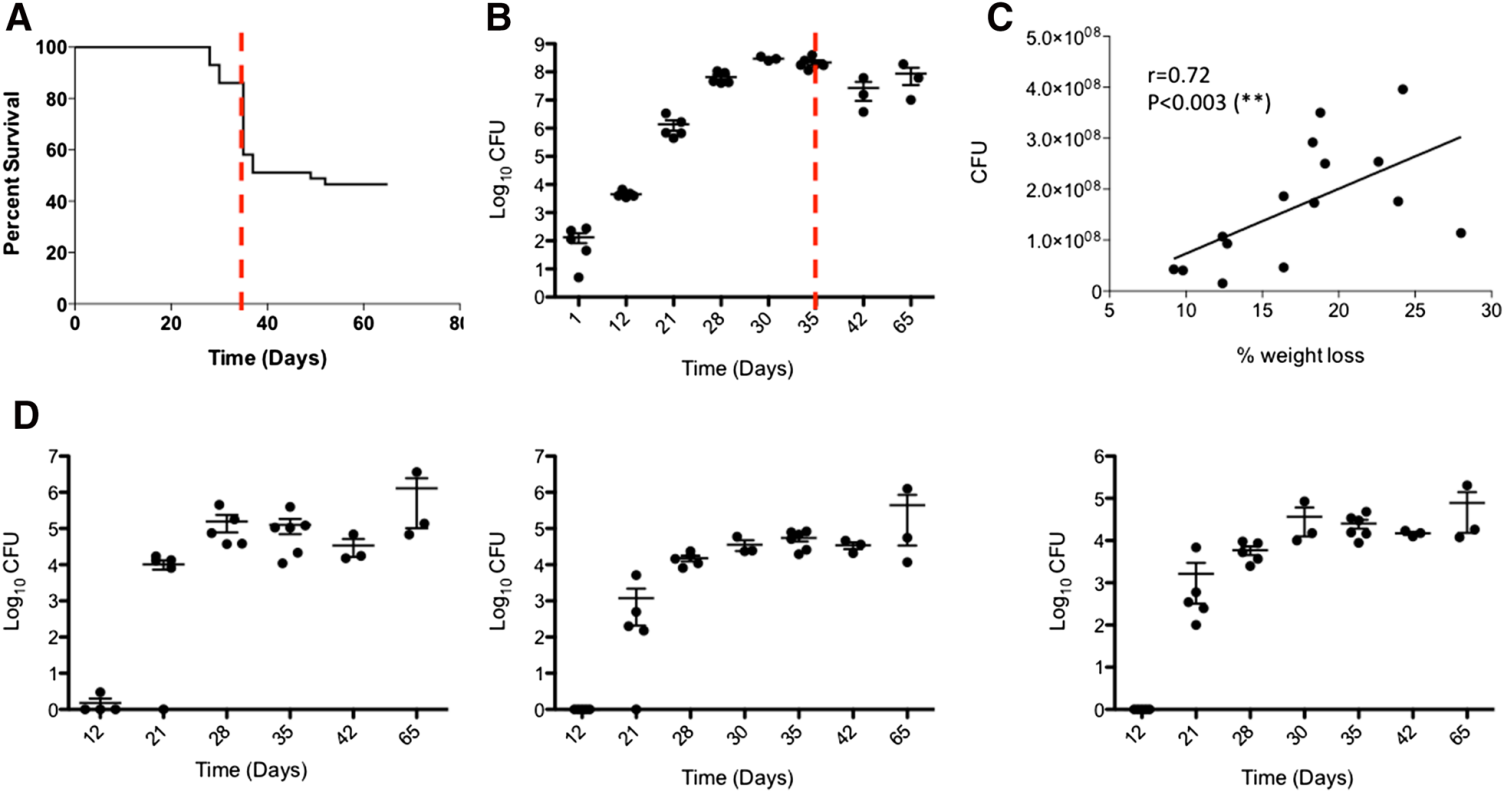
**Kinetics of bacterial replication of** ***M. bovis*** **within C3HeB/FeJ mice and associated morbidity.** **A** Survival curve of C3HeB/FeJ mice following intranasal infection with *M. bovis* AF2122/97 (*n* = 43). The dotted red line delimitates the critical early phase of infection (week 5). **B** Bacterial growth curve in the lung of C3HeB/FeJ mice following intranasal infection. Data are presented as mean log_10_ CFU ± s.e.m (*n* = 5 to 8 mice per time point). The dotted red line delimitates the critical early phase of infection (week 5). **C** Focus on the early phase of infection (0 to 5 weeks) showing the positive correlation of animal total body weight loss with pulmonary bacterial burden (Spearman’s correlation indexes: r = 0.72 & **p < 0.003). End point body weight was taken prior euthanasia and compared to the maximal weight of the animal during the course of the experiment and lung bacterial load are expressed as log_10_ CFU. Mice were euthanized between 21 and 35 days post-infection (*n* = 15). **D** Bacterial burden in lung-draining lymph node (left graph), spleen (middle graph) and Liver (1 lobe, right graph) of C3HeB/FeJ mice following intranasal infection. Data are presented as mean log_10_ CFU ± s.e.m (n = 5 to 8 per time point).

C3HeB/FeJ mice were intranasally infected with 200 colony-forming units (CFU) of *M. bovis* AF 2122/97 and followed for weight loss and morbidity symptoms until terminal endpoints. A regular window of mortality was observed between 4 and 5 weeks post-infection where 40 to 60% of total mice, depending on the experiments, had to be sacrificed due to extensive and rapid weight loss (>15% in 3 days) associated with morbidity symptoms (e.g. lethargy, ruffled fur) (Figure 2A). Mice that got through this critical period constantly survived at least 9 weeks (end point of our study) with no obvious or minimal clinical signs (Figure 2A). These observations of incidence of a group of early-mortality mice are consistent with previous results by Irwin and al. in C3HeB/FeJ mice infected with the *M. tuberculosis* Erdman strain [31]. As a comparison, C57BL/6 mice intranasally infected with 200 CFU of *M. bovis* AF 2122/97 didn’t show any weight loss or any morbidity symptoms during the 9 weeks period (data not shown).

The survival curve of the C3HeB/FeJ mice following infection with its windows of early mortality coincides with the fast bacterial replication in the lung reaching a plateau of 8 log10 CFU by 30 days post-inoculation (Figure 2B).

In order to investigate the critical early phase leading to morbidity in a number of mice, we compared pulmonary bacterial load with the percentage of total body weight loss of individual mice at the time of euthanasia between 3 and 5 weeks (Figure 2C). Total body weight loss significantly correlated with pulmonary bacterial load, thus providing a reliable parameter, in addition to morbidity symptoms to predict early disease severity (Spearman’s correlation indexes: r = 0.72 and *p* < 0.003).

Next we studied the kinetics of disease progression and detected bacilli as early as 12 days post-infection within the tracheobronchial draining lymph nodes reaching around 6 log_10_ CFU by 65 days post-inoculation (Figure 2D, left panel). By 21 days post-infection the bacilli started to spread to extra-pulmonary tissues such as spleen and liver reaching a plateau just below 5 log_10_ CFU by day 35 (Figure 2D, middle panel) and 30 (Figure 2D, right panel), respectively. Similarly to the lung, the bacterial numbers in these tissues increased until 30–35 days post-inoculation tended to reach a plateau and then slightly increased by 65 days (Figure 2D).

### Development of Type I, II and III pulmonary lesions by C3HeB/FeJ mice over the course of *M. bovis* infection

**Figure 3:**
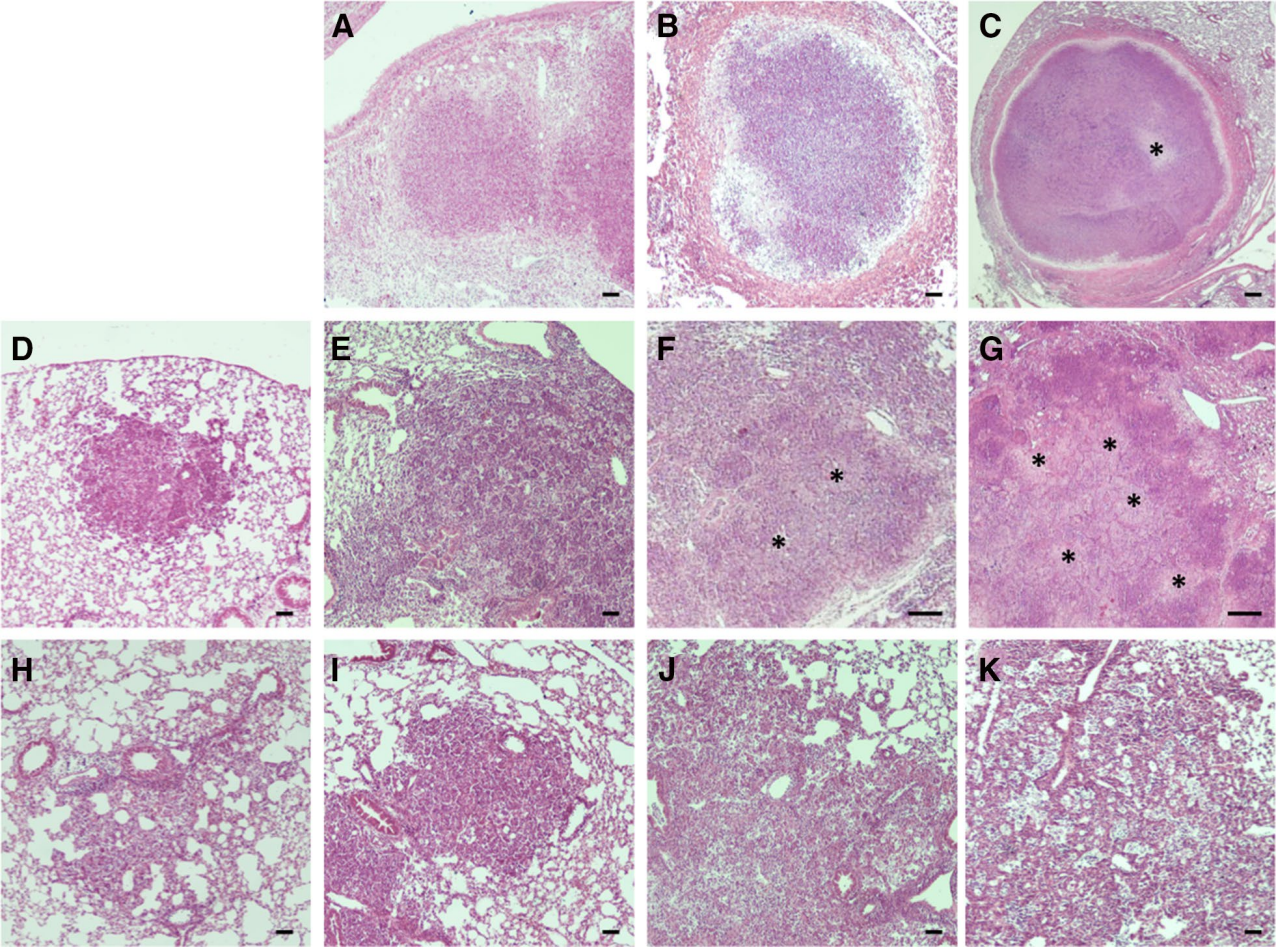
**Development of Type I, II and III lesions in lung of C3HeB/FeJ mice over** ***M. bovis*** **course of infection.** Each panel represents a lesion (H&E) obtained from individual representative C3HeB/FeJ mice euthanized at different time-points following *M. bovis* AF2122/97 intra-nasal infection. **A–C** Progression of Type I lesions from animals at day 35 (**A**), 42 (**B**) and 65 (**C**, “*”: caseous necrosis). **D–G** Progression of Type II lesions from animals at day 21 (**D**), 23 (**E**), 28 (**F**) and 37 (G, “*”: caseous necrosis). **H–K** Progression of Type III lesions from animals at day 21 (**H**), 28 (**I**), 42 (**J**) and 65 (**K**). Scale bars: 100 μm (**A**, **B**; **D**–**K**); 200 μm (**C**).

Systematic histopathological analysis of the whole lung of C3HeB/FeJ mice revealed lesions in various stages of development occurring simultaneously. Three distinct lesion types were clearly apparent by 5 weeks post-infection in accordance with Irwin et al. classification [31]. Type I lesions (Figures 3A–C) corresponded to the classical encapsulated TB granulomas with a caseous necrotic core. Type I lesions resulted from the occlusion of alveolar spaces by a cellular infiltrate that evolved into an organized granuloma visible 5 weeks post-infection with a central accumulation of foamy macrophages and neutrophils surrounded by epithelioid macrophages interspersed by few lymphocytes while, in periphery, the fibrotic rim was not yet fully established (Figure 3A and Additional file 1A). After 6 weeks the neutrophil core increased with adjacent clusters of neutrophils, foamy and epithelioid macrophages while the periphery was composed of a rim of diffuse foamy macrophages underneath a rising fibrous capsule (Figure 3B and Additional file 1B). After 7–9 weeks Type I granulomas continued to extend without significant evolution of cellular composition but increased associated fibrosis from small collagen fibers to form a thick fibroblast-rich ring while the neutrophilic core continued to degenerate into karyorrhectic debris and further caseous necrosis (Figure 3C). Later on, we observed cavitary lesions as described following *M. tuberculosis* infection [32].

Type II lesions appeared earlier than Type I, being clearly distinguishable 3 weeks post-inoculation, with a fast progression reaching a climax 5 weeks post-infection (Figures 3D–G). These rapidly progressive lesions started as a granulocytic pneumonia mostly composed of neutrophils emerging 3 weeks post-infection (Figure 3D and Additional file 1C). Type II lesions further quickly derived into invasive caseous necrotic pneumonia between 3 and 4 weeks post-inoculation (Figures 3E–F). At this point the lesion evolved with the emergence of very large multicentric caseous necrotic areas composed of cellular necrosis and karyorrhectic debris (mostly of neutrophil origin), but earlier and faster than Type I lesions (Figure 3G and Additional file 1D). Furthermore, as opposed to Type I lesions, Type II lesions did not include organized encapsulating fibrotic rim (Figure 3G).

Type III lesions were clearly visible from week 3 post-infection proximally to large blood vessels and bronchioles and were mostly composed of activated macrophages and lymphocytes with only few detectable neutrophils (Figure 3H and Additional file 1E). Between 4 and 5 weeks post-infection large numbers of epithelioid and foamy macrophages were visible interspersed with lymphocytes (Figures 3I and J, and Additional file 1F). Further, most of the macrophages observed in those pneumocytic lesions transformed into foamy macrophages, displaying numerous lipid vesicles and forming small cavities containing karyorrhectic debris (Figure 3K). Type III lesions corresponded to the cellular inflammatory “cuffy” pneumonia infiltrate typically observed in TB resistant mouse models, such as BALB/c and C57BL/6 following *M. tuberculosis* infection [19, 20]. In a similar way, C57BL/6 mice that were included in some of our experiments developed only Type III lesions during the course of infection following intranasal inoculation with 200 CFU of *M. bovis* AF 2122/97 (Additional file 2).

### C3HeB/FeJ Type I pulmonary lesions recapitulate the features of cattle lung granuloma

**Figure 4:**
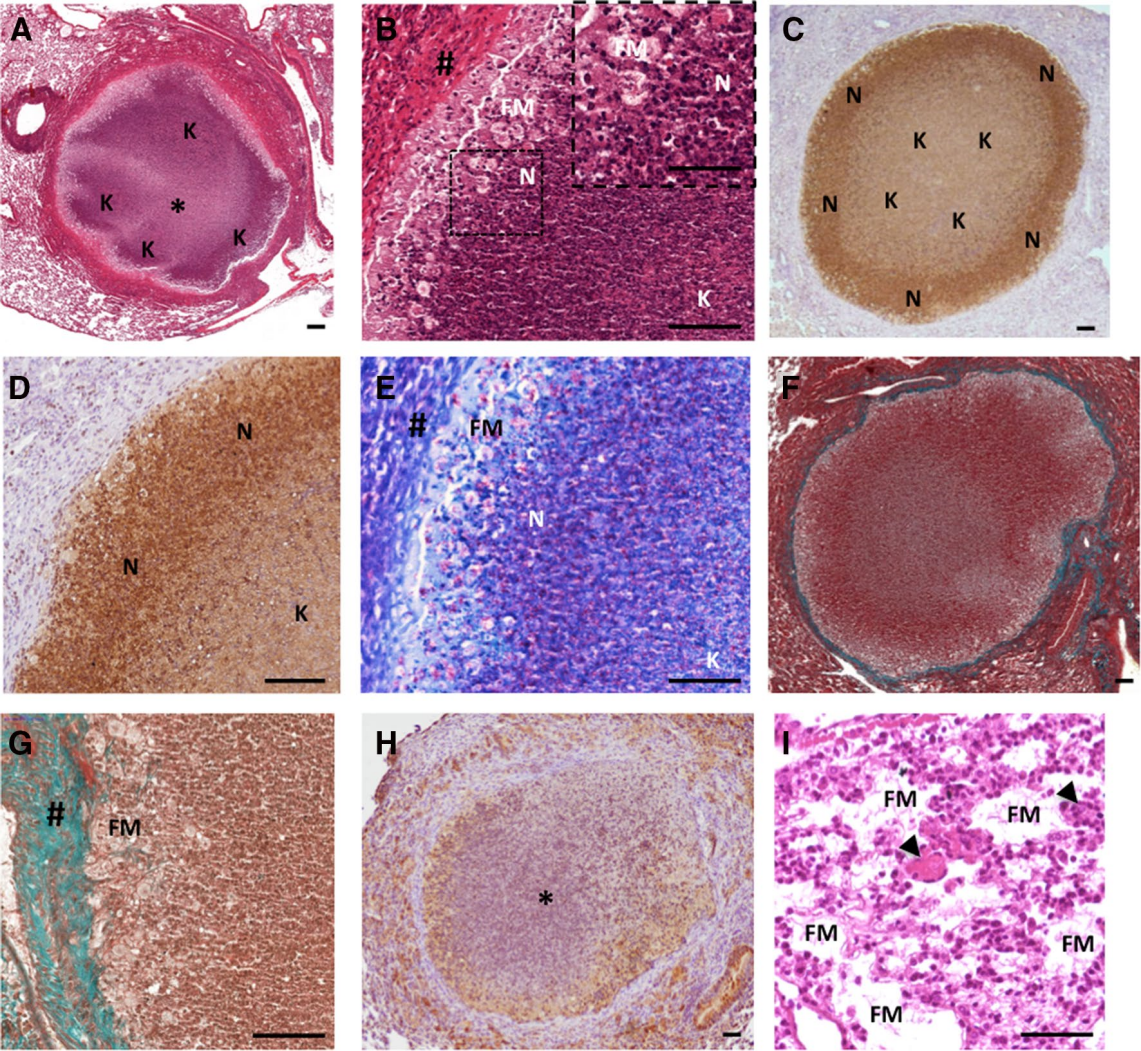
**The C3HeB/FeJ Type I lesions recapitulates the features of the stage III/IV cattle granuloma lesions.** **A** H&E staining of a Type I granuloma showing acellular caseum (*) surrounded by typical neutrophil-derived karyorrhectic debris areas (“K”). **B** Higher magnification of A showing a characteristic dark stained neutrophil dense area (“N” & Zoom) next to the foamy macrophages ring (FM) below the fibrous capsule (#). **C**,** D** Immunohistochemical staining with Ab against Ly-6G (clone 1A8) showing the high density of neutrophil within the subscapular area (“N”) and neutrophil-derived karyorrhectic debris within the core of the lesion area (“K”). **E** Ziehl staining of the same area as B showing peripheral cellular bacilli within foamy macrophages (“FM”) and neutrophil (“N”) and central acellular bacilli within the karyorrhectic debris (“K”). **F**,** G** Masson-Goldner’s Trichrome staining showing the collagen capsule all around a Type I lesions (**F**). **G** Higher magnification of F showing the collagen staining among the fibroblast in top of a rime of foamy macrophages. # fibrous capsule, FM foamy macrophages. **H** Pimonidazole staining showing characteristic hypoxia within central necrotic granuloma (*) and its periphery. **I** H&E staining, high magnification of foamy macrophages (“FM”) and epitheliod cell dense area containing multinucleated Langhans giant cells (black arrows). Scale bars: 50 μm (B-Zoom, I); 100 μm (**A**–**H**).

Detailed examination of Type I pulmonary lesions in C3HeB/FeJ (Figure 4) highlighted their close resemblance to the classical lung granuloma observed in aerogenous, experimentally or naturally infected cattle (Figures 1A–D, Table 1). They were composed of a central acellular caseum, interspersed with karyorrhectic debris derived from necrotic neutrophils, surrounded by scattered intact neutrophils close to a rim of foamy macrophages underneath the fibrous capsule (Figures 4A–D). Mycobacteria staining confirmed the presence of numerous acellular bacilli within the necrotic central areas and intra-cellular bacilli mostly within foamy macrophages at the periphery (Figure 4E). Masson-Goldner’s Trichrome staining for collagen clearly highlighted the circumscribing fibrotic rim while pimonidazole staining confirmed the presence of a characteristic hypoxic environment around and within these necrotic granulomas (Figures 4F–H), as previously described [21]. Furthermore, high magnification observation of the pulmonary lesions allowed the detection of typical multinucleated Langhans giant cells scattered within foamy macrophages dense areas as observed within cattle lesions (Figure 4I vs. Figure 1I).

### Relevance of Type II and III C3HeB/FeJ lesions compared to cattle lesions

**Figure 5:**
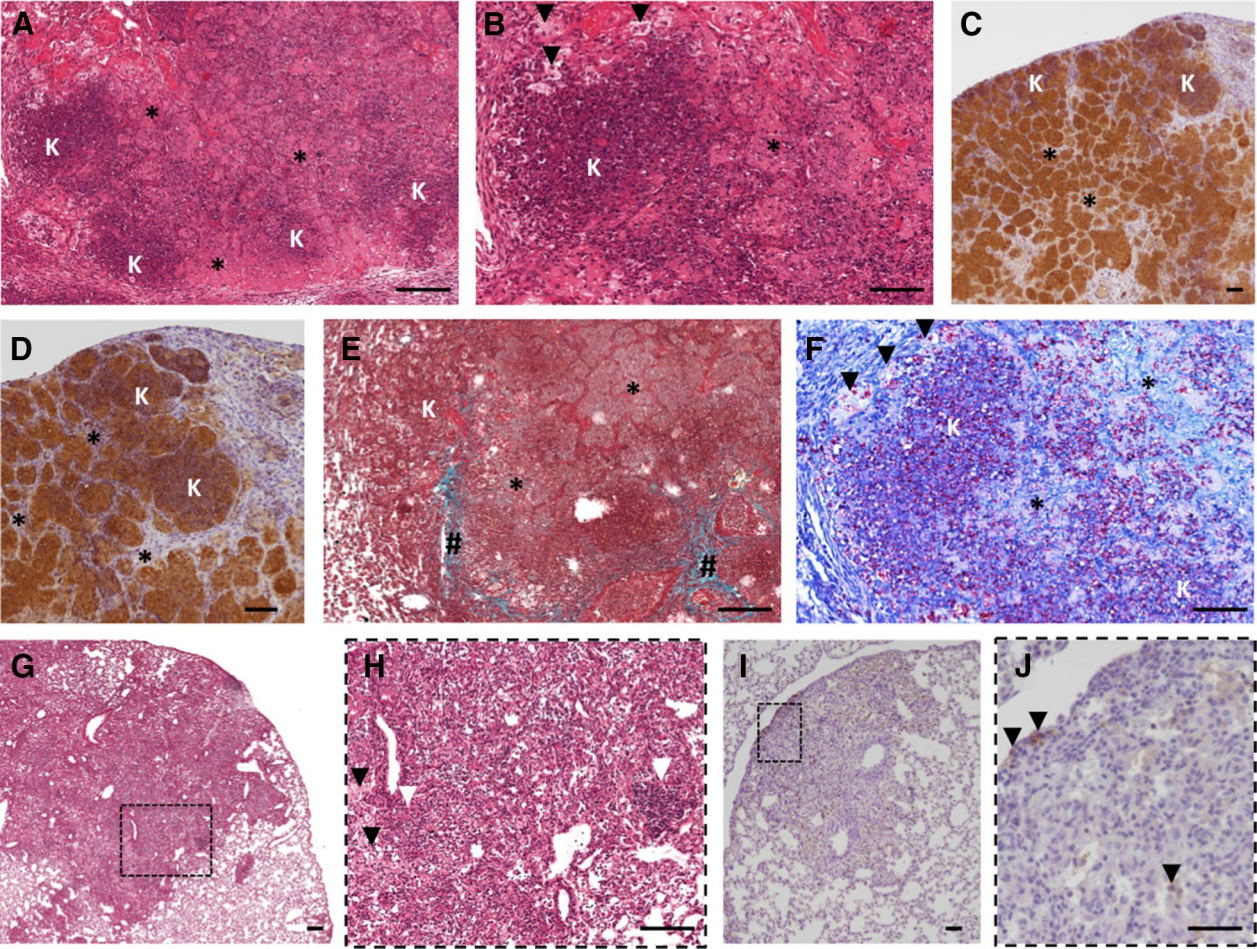
**Characterization of Type II and Type III lesions.** (**A**–**F**) Type II lesions. **A** H&E staining of a typical multicentric bubble shaped Type II lesion showing pulmonary acini totally occluded with neutrophils and karyorrhectic debris. “K”: karyorrhectic debris, “*” necrosis area. **B** Higher magnification of A showing necrosis areas (*) surrounded by neutrophilic karyorrhectic debris (“K”) interspersed by foamy macrophages (black arrows). **C**, **D** Immunohistochemical staining with Ab against Ly-6G (clone 1A8) showing the dense neutrophilic composition of the multicentric bubble shaped Type II lesion with aggregates of neutrophil-derived karyorrhectic debris (“K”). **E** Masson-Goldner’s Trichrome staining of the same area as A showing few scattered discontinuous fibrous capsule areas (#). **F** Ziehl staining of the same area as B showing dense acellular bacilli concentration within the karyorrhectic debris (“K”) and the necrotic areas (*) surrounded by bacilli-rich foamy macrophages (black arrows). **G–I** Type III lesions. **G**, **H** H& E staining of Type III lesions showing a characteristic perivascular and peribronchial inflammatory infiltrate, typical of a lymphocytic “cuffy” pneumonia, composed of foamy macrophages (black arrows) interspersed with epitheliod cells and a large number of lymphocytes scattered or in cluster (white arrow). **I**, **J** Immunohistochemical staining with Ab against Ly-6G (clone 1A8) showing the low representation of neutrophils within Type III lesion infiltrate (black arrows: neutrophils). Scale bars: 50 μm (**B**, **F**, **G**, **J**); 100 μm (**A**, **C**, **D**, **E**, **H**, **I**).

Type II lesions displayed multiple karyorrhectic areas of degenerating neutrophils in caseous necrotic ranges (Figures 5A–D) interspersed with foamy macrophages (Figure 5B). Besides a higher density of intact and degenerated neutrophils as compared to Type I lesions, Type II lesions also exhibited low discontinuous fibrotic structure as shown by Masson-Goldner’s Trichrome staining (Figure 5E). Strikingly, Ziehl staining within Type II lesions reveal an extensive bacterial load within the neutrophil multiple necrotic and caseous areas (Figure 5F), in accordance with the exacerbated lung lesions observed in asymptomatic cattle naturally or experimentally infected with *M. bovis* [23, 25]. Thus the rapidly growing Type II lesions observed within lungs of C3HeB/FeJ following *M. bovis* infection shared all the features of the large multicentric caseous lesions, described as invasive necrotizing granuloma [30], observed in cattle (Figures 1E–H, Table 1).

The Type III lesions, characterized by large perivascular and peribronchial areas of foamy macrophages and lymphocytes (scattered or in clusters) and few scattered neutrophils (Figures 5G–J) were clearly reminding of chronic lymphocytic “cuffing” pneumonia, with low mycobacterial load, described within early pulmonary lesions in infected calves ([23, 24], Table 1).

### Pulmonary lesion burden and heterogeneity following *M. bovis* infection in C3HeB/FeJ mice

**Figure 6:**
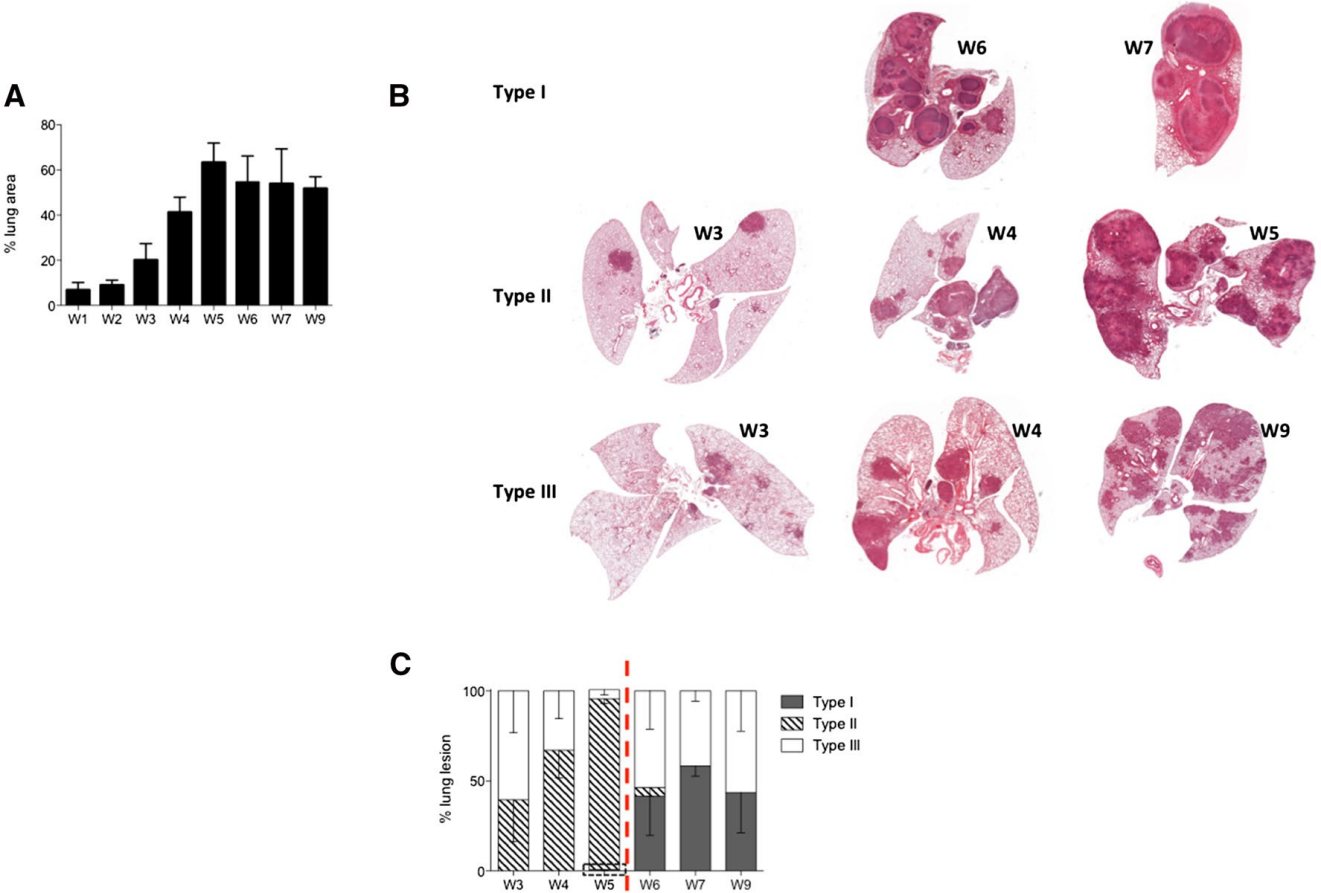
**Progression of total lung lesion burden and Type heterogeneity following the course of** ***M. bovis*** **infection.** **A** Morphometric analysis of the total lesion area performed on H&E-stained sections of the global lung (5 lobes) of mice following intranasal infection with *M. bovis*. Histograms represent the mean percentage of lesion area within the total lung ± s.e.m at 1 to 9 weeks post-infection (*n* = 5 to 8 mice per time point). **B** High resolution scanned slides of H&E stained lung from representative C3HeB/FeJ mice at different weeks post-infection (W3 to W9) showing the characteristic evolution of Type I (top images), Type II (middle images) and Type III (down images) lesions over the course of infection. **C** Histometric analysis of the relative percentage of each Type of lesion within the total lesion area performed on H&E-stained sections of the global lung (5 lobes) of mice following intranasal infection with *M. bovis*. Histograms represent the mean percentage of each lesion Type over the total percentage (100%) of lesion area ± s.e.m at 1 to 9 weeks post-infection (*n* = 5 to 8 mice per time point). The dotted rectangle indicate the presence, at a low rate (0.7%), of emerging Type I lesions as soon as 5 weeks post-infection. The dotted red line delimitates the critical early phase of infection (week 5).

Morphometric analysis of the total lung area occupied by lesions was performed using an Image-J macro on H&E-stained histological extensive sections of the total lung from individual C3HeB/FeJ collected between week 1 and 9 (*n* = 3–8 mice per collecting time). First evidence of tuberculous lesions occurred 2 weeks after infection and rapidly increased between week 3 and 5 where it reached a peak with about 63% of the lung area affected before stabilizing with no significant increase further measured (Figure 6A). These data agreed with the bacterial load progression that we observed in the lung reaching a plateau between 4 and 5 weeks post-infection (Figure 2B) and the survival curve depicting only minor mortality occurring between week 5 and 9 (Figure 2A).

These combined data highlighted two orientations for the pathology in the lung of C3HeB/FeJ, as well represented by the survival curve (Figure 2A), with a critical early phase between weeks 4 and 5 where 40 to 60% mice (depending on the experiment) developed acute lethal pneumopathy while others followed a more progressive pathology with no obvious clinical signs until 9 weeks, the end-point of our study.

To complete this global lung lesion measurement we performed a systematic pathological analysis on scanned histological slides to quantify the global occurrence of each lesion type during the course of infection (see examples in Figure 6B). It is important to note that this histological analysis was influenced by the increased occurrence of mice reaching terminal endpoints between week 4 and 5. Nevertheless, the normalization of each lesion type percentage among total lung burden recapitulated the global histological development of the pathology depicting again bimodal evolution with the rapid increase of the early Type II lesions followed by a stabilized pathology with more balanced between Type I and III lesions (Figure 6C).

### Type II lesions are associated with a higher pulmonary bacterial load and body weight loss responsible for early mortality

**Figure 7:**
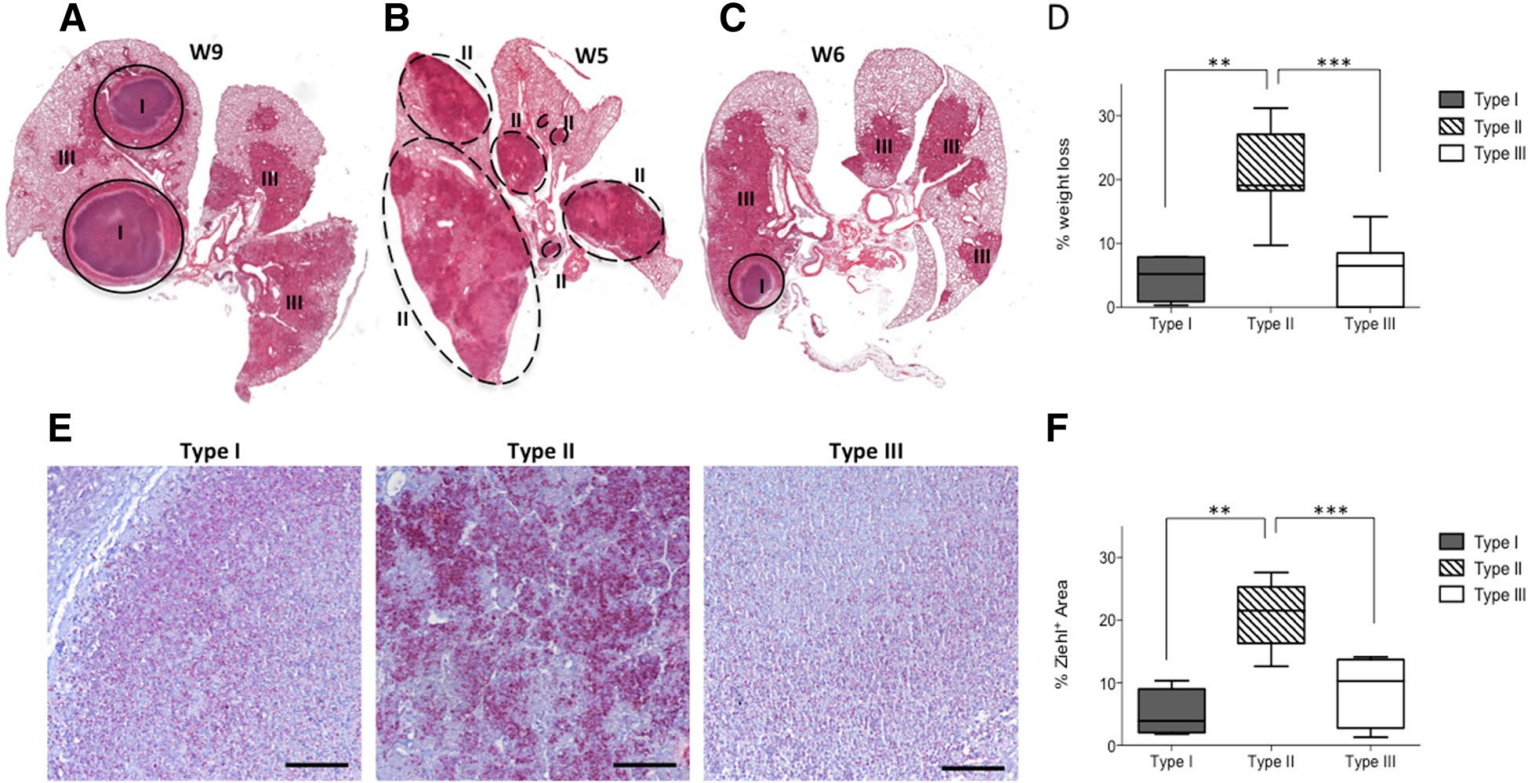
**Type II lesions are associated with a higher pulmonary bacterial load inducing body weight loss and early mortality.** **A**–**C** High resolution scanned slides of H&E stained lung from representative individual C3HeB/FeJ mice showing the intra-mouse lung lesions heterogeneity following infection. **A** Total lung showing typical Type I and Type III lesions at 9 weeks post-infection. **B** Total lung lobes showing only Type II lesions at 5 weeks post-infection. **C** Total lung lobes showing the presence of Type III and Type I lesions at 6 weeks post-infection. **D** Link between total body weight loss and Type of lesions present in the lung of the mice following infection. Analysis was conducted all over the course of infection (Between weeks 3 and 9) considering the most represented Type of lesion within the total lung of each individual (*n* = 26). Statistical comparison performed using Mann–Whitney’s test. **p = 0.005, ***p = 0.0001 **E** Representative Ziehl staining of advanced Type I, II & III lung lesions showing the bacterial density and repartition. Scale bars: 100 μm. **F** Link between bacterial density (% of Ziehl positive area) and Type of lesions present in the lung of the mice following infection. Analysis was conducted all over the course of infection (Between weeks 3 and 9) considering representative lesions from individual mice (*n* = 23). Statistical comparison was performed using Mann–Whitney’s test. **p = 0.005, ***p = 0.0001.

Systematic histological observation of intra-mouse lung lesions heterogeneity throughout infection pointed out different patterns of lesion types repartition. Indeed development of Type II lesions, that rapidly spread within all the lung lobes, was quite exclusive in affected mice that rapidly succumbed, (Figure 6B middle panel and Figure 7B), while in surviving animals either a majority of Type III lesions (Figure 6B, down panel) or Type I lesions (Figure 6B, upper panel) or a mix of Type I and III lesions was observed (Figures 7A and C). These first observations were confirmed by the quantification by histometrical analysis of the percentage of each Type of lesions within the total lesion area during the course of infection (Figure 6C). Therefore, these data reinforced the concept of a dichotomy in the pathology with some mice exclusively drawn into the fast and deleterious Type II pathway while others followed a more progressive evolution with Type I and Type III lesions. In addition, this result was supported by combining, for each individual mice, the full-body weight loss data with the lesion types. This showed a statistically significant positive correlation between weight loss and the presence of Type II lesions (Figure 7D). Detailed and quantified analysis of the Ziehl-stained histological lung sections highlighted the higher density of bacilli present within Type II lesions (illustrated in Figure 7E) and further confirmed the positive correlation between pulmonary bacterial load and the occurrence of Type II lesions (Figure 7F).

By linking those data with the CFU vs weight loss significant correlation observed during the early phase (Figure 2C) we could clearly state that Type II lesions development was responsible for the early mortality, occurring between Weeks 4 and 5 following infection, in a significant percentage of the infected mice (Figure 2A).

## Discussion

This study, which represents the first characterization of the response of the C3HeB/FeJ mouse model to *M. bovis* infection, clearly establish the potential of the Kramnik’s mouse model to closely replicate the specific hallmarks of bTB in the natural target species: the cow [22]. Indeed our results show that, following intranasal infection with *M. bovis*, C3HeB/FeJ mice reproduce the histopathology that we and others observed in the lungs of naturally or experimentally infected cattle (Figure 1, Table 1 [23–25]).

Moreover, we clearly distinguished three different types of lesions developing in the lungs of C3HeB/FeJ following *M. bovis* (AF2122/97) infection, in accordance with the recent work from Irwin et al. with *M. tuberculosis* (Erdman) [31].

Type I lesions are clearly share all the features of the classical granuloma reported in the lungs of naturally and experimentally infected cattle (Table 1).

Type II lesions represent rapidly progressive granulocytic pneumonia compromising the survival of the mice. Even though these lesions share features with Type I lesions, they quickly evolve in multicentric necrotic lesions composed of degenerated neutrophils. More importantly, Type II lesions do not develop an efficient granuloma encapsulation process with only rare and scattered fibrotic areas. Moreover, Type II lesions are characterized by a cellular composition strongly oriented towards neutrophils and display the highest bacilli loads. All these histopathological features are reminiscent of the invasive necrotizing granulomas also reported in the lung of cattle naturally and experimentally infected (Table 1). However these type of lesions were found in the lung of asymptomatic cattle following natural infection with *M. bovis*, without any report of impact on weight loss or survival [25], which differs from the mouse model.

Type III lesions represent typical inflammatory “cuffing” pneumonia infiltrates similar to the one observed following *M. tuberculosis* inoculation in TB resistant mouse models such as BALB/c and C57BL/6 [19, 20]. Neutrophils are extremely rare and the amount of bacteria detected in Type III lesions is low. All these features are in accordance with the chronic lymphocytic “cuffing” pneumonia reported in the lung of cattle naturally and experimentally infected (Table 1).

The rapid arising of Types II lesions occurring in 40 to 60% of the C3HeB/FeJ, is responsible of a striking phenomenon of early mortality This terminal endpoint was also reported by Irwin et al. after *M. tuberculosis* infection, but in lower percentage of mice (20 to 40%) [31]. This notable difference is in accordance with several reports pointing out the higher “virulence” (i.e. bacterial burden and/or mortality) of *M. bovis* vs *M. tuberculosis* following experimental infection in several mouse models or rabbits ([33, 34]).

One critical aspect of the histopathology of *M. bovis* infection in C3HeB/FeJ mice resides in the key role of neutrophils in the orientation, organization and dynamics of the lesions. Indeed the severity of the lesions correlates with the amount of neutrophils as already described by Major et al. in CBA/J mice following *M. tuberculosis* infection. This similar bimodal evolution of the pathology was reported in CBA/J mice before it was spotted by Irwin et al. in C3HeB/FeJ mice, and Niazi et al. in Diversity Outbred mice, all in the context of *M. tuberculosis* infection [31, 35, 36]. Moreover Marzo et al. realized a detailed demonstration of the damaging role of the neutrophil infiltration within the C3HeB/FeJ mouse model following high dose of *M. tuberculosis* infection [37]. Therefore, it clearly appears that mice susceptible to TB, as reported by others, and also to bTB, as our data illustrate, develop highly neutrophil-oriented lesions. Strikingly, the localization pattern of neutrophils and mycobacteria, as observed by staining of the lesions, seems really similar with a high density of neutrophils and mycobacteria in the periphery of Type I lesions (Figures 4D and E) and a global higher density with numerous patch rich in neutrophil and mycobacteria within Type II lesions (Figures 5D and F). Moreover the positive correlation between neutrophil counts in lung lesions and bacterial load was also reported in cattle naturally infected with *M. bovis* by the aerogenous way, with a higher abundance of those cells within lesions similar to Type II ([25], Table 1). Our observations on naturally infected cattle also supported these reports, even though we did not quantify neutrophils, either intact or degenerated. Taken together those data clearly reinforce the concept of a detrimental role of neutrophils in the course of TB infection in cattle. Actually this concept, also reported in the human pathology, is now even considered as a potential leverage for human TB therapy [16]. Moreover they strongly support the biological relevance of the Type II lesions occurring following early events at the host-bacteria interface and not related to some experimental bias of the C3HeB/FeJ model as previously hypothesized [31].

It is likely that the role of neutrophils during bTB has been underestimated. The stage of evolution of bovine granuloma, as settled by Wangoo et al. depicts a minimal representation of neutrophils within the dynamic organization of lymph nodes lesions [38]. Interestingly, other reports examining lung lesions from (i) experimentally intranasally infected [23], (ii) infected by experimental contact with reactors [24], or (iii) naturally infected cattle herds [30, 25], all highlight the importance of the neutrophil infiltrate and degeneration in the lung compartment participating in the formation of the necrotic core [23], as we observed in the C3HeB/FeJ mouse model. Furthermore, Menin et al. in their study of 247 naturally *M. bovis*-infected cattle also established a strong positive correlate between lung bacterial load, lesion severity score and neutrophil abundance, while the level of encapsulation negatively correlated with the severity score [25]. These data in cattle fully agree with our results in the C3HeB/FeJ model with the same parameters distinguishing Type I from Type II lesions. Moreover, several reports from lung lesions collected on free-ranging wildlife support the importance of neutrophils in the pulmonary pathology with their abundance within necrotic lesions in elk/red deer and fallow deer while they remain more sparse in lymph nodes [30]. Likewise, a study on naturally infected badgers also reported the presence of lung lesions strikingly remaining of the invasive necrotizing granulomas within cattle (Table 1) and the Type II lesions that we observed in the C3HeB/FeJ [39].

All these elements are clearly in favor of a potential specificity of the lung environment compared to the lymph nodes regarding the role of the neutrophils in the development of the tuberculous lesions.

To conclude, we report here that the C3HeB/FeJ mouse model recapitulates the histopathological features of bTB following aerogenous *M. bovis* infection Assuming the biological relevance of this type of lesions resembling those observed in cattle and wildlife, we suggest placing Irwin et al. classification, especially regarding the dichotomy between Type I and type II lesions, in the dynamics context of the lung lesions development. In this manner, early events within the alveolar compartment that control the level of neutrophil recruitment, the level of fibrotic encapsulation and the bacterial burden might further lead to either invasive or controlled lesions. In this context the C3HeB/FeJ mouse represents a relevant model for bTB pathophysiology and also shows promises for the preclinical development of vaccine strategies for cattle, which are in deep need if we want to efficiently control this worldwide zoonosis.

## Additional files

**Additional file 1.** **Immunohistochemical staining of neutrophils using Ab against Ly-6G (clone 1A8) on C3HeB/FeJ lung lesions.** (A-B) Type I lesions. (C-D) Type II lesions. (E–F) Type III lesions, black arrow indicates scattered neutrophils. Scale bars: 50 μm (C, E); 100 μm (A, B, D, F).

**Additional file 2.** **H&E staining of lesions within lungs of C57BL/6 mice: 5** **weeks (A) and 7** **weeks (B) following intranasal infection with 200** **CFU of** ***M. bovis*** **AF 2122/97.** Lesions are typical of Type III with a characteristic perivascular and peribronchial inflammatory infiltrate mostly composed of lymphocytes interspersed with foamy macrophages. Scale bars: 100 μm.

## Abbreviations

bTB: bovine tuberculosis
DIVA: distinguish vaccinated from infected animals
TB: tuberculosis
MGC: multinucleated Langhans giant cell
H&E: haematoxylin and eosin
AFB: acid-fast bacilli
ZN: Ziehl–Neelsen
CFU: colony forming units
