# Supplementary figures and images for: The C3HeB/FeJ mouse model recapitulates the hallmark of bovine tuberculosis lung lesions following Mycobacterium bovis aerogenous infection
Source: Vet Res. 2017 Nov 7;48:73. doi: 10.1186/s13567-017-0477-7 (PMC5678586; doi:10.1186/s13567-017-0477-7)

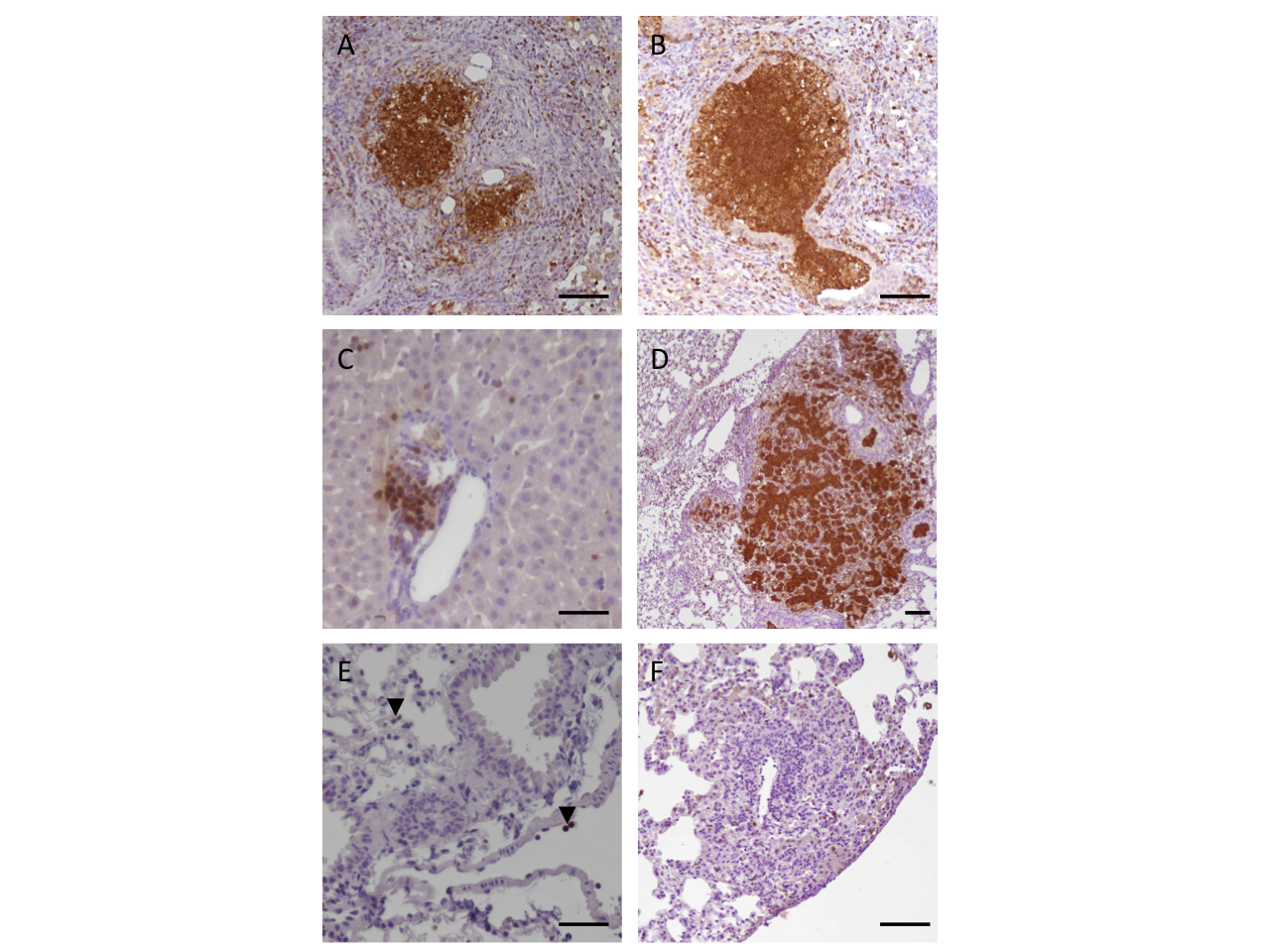

Supplement: Supplementary file 1 — Additional file 1. Immunohistochemical staining of neutrophils using Ab against Ly-6G (clone 1A8) on C3HeB/FeJ lung lesions. (A-B) Type I lesions. (C-D) Type II lesions. (E–F) Type III lesions, black arrow indicates scattered neutrophils. Scale bars: 50 μm (C, E); 100 μm (A, B, D, F). [file 13567_2017_477_MOESM1_ESM.tiff]

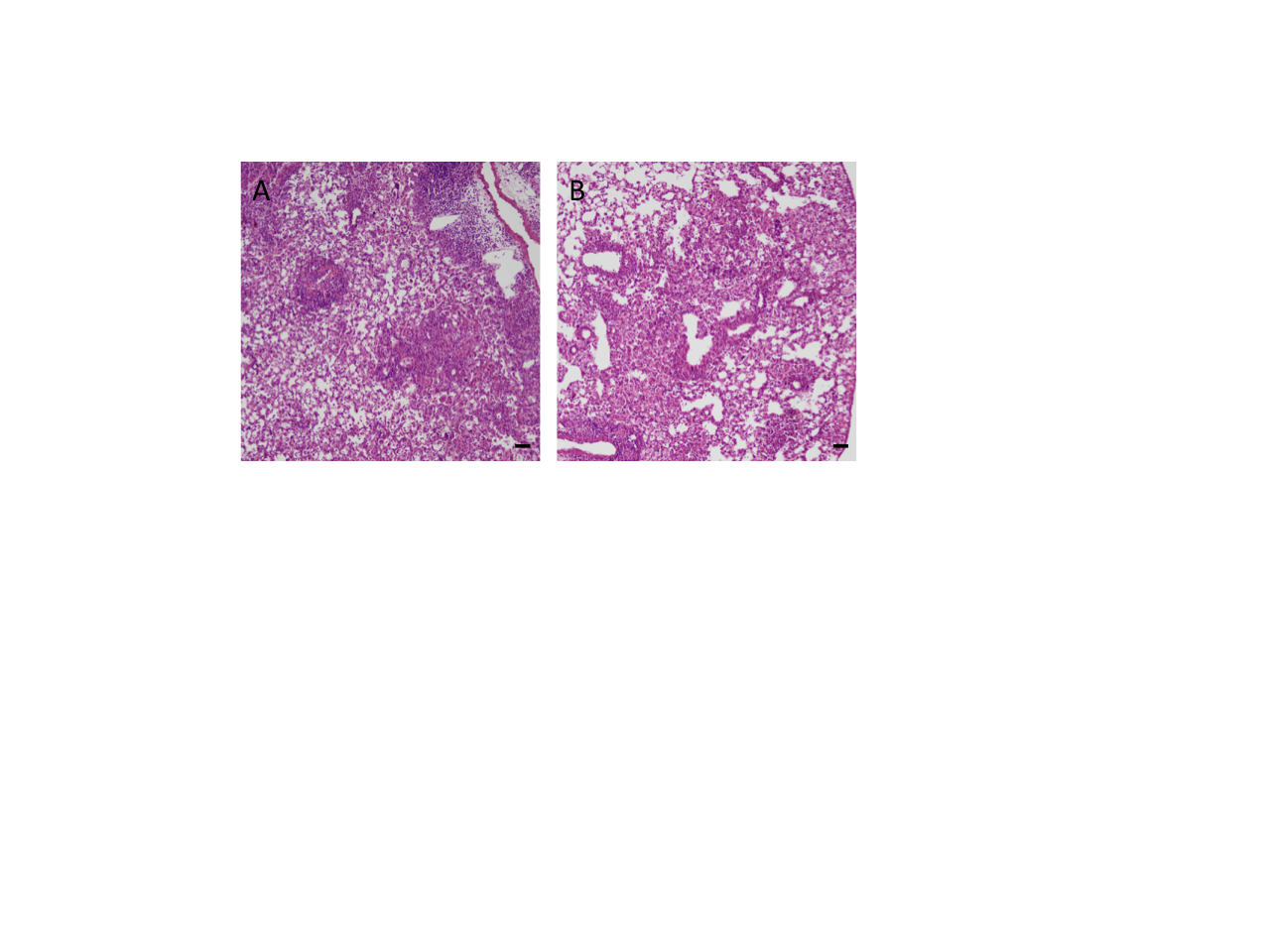

Supplement: Supplementary file 2 — Additional file 2. H&E staining of lesions within lungs of C57BL/6 mice: 5 weeks (A) and 7 weeks (B) following intranasal infection with 200 CFU of M. bovis AF 2122/97. Lesions are typical of Type III with a characteristic perivascular and peribronchial inflammatory infiltrate mostly composed of lymphocytes interspersed with foamy macrophages. Scale bars: 100 μm. [file 13567_2017_477_MOESM2_ESM.tiff]
